# A novel therapy for eliminating phrenic nerve stimulation: a long-term follow-up case report

**DOI:** 10.3389/fcvm.2026.1700683

**Published:** 2026-03-13

**Authors:** Jia Li, Hongwei Yi, Hongwei Han, Hua Yan

**Affiliations:** Department of Cardiology, Wuhan Asian Heart Hospital, Wuhan, Hubei, China

**Keywords:** cardiac resynchronization therapy (CRT), phrenic nerve stimulation (PNS), phrenic nerve (PN), heart failure (HF), left ventricular (LV), coronary sinus (CS)

## Abstract

Phrenic nerve stimulation (PNS) is a frequent occurrence in patients implanted with a cardiac resynchronization therapy (CRT) device. Most PNS can be eliminated by external programming of pacing threshold, pacing polarity, etc., but there are still a small number of refractory PNS cases that need electrode reset. The latter has many uncertainties, such as poor coronary sinus (CS) target vessel conditions, venous pathway obstruction or occlusion, recurrence of PNS, pacemaker sac infection, disappearance of CRT super response, etc. In view of this, we reported one case of PNS after CRT treated by isolating the left phrenic nerve (PN) and the left ventricular (LV) electrode.

## Introduction

**Figure 1:**
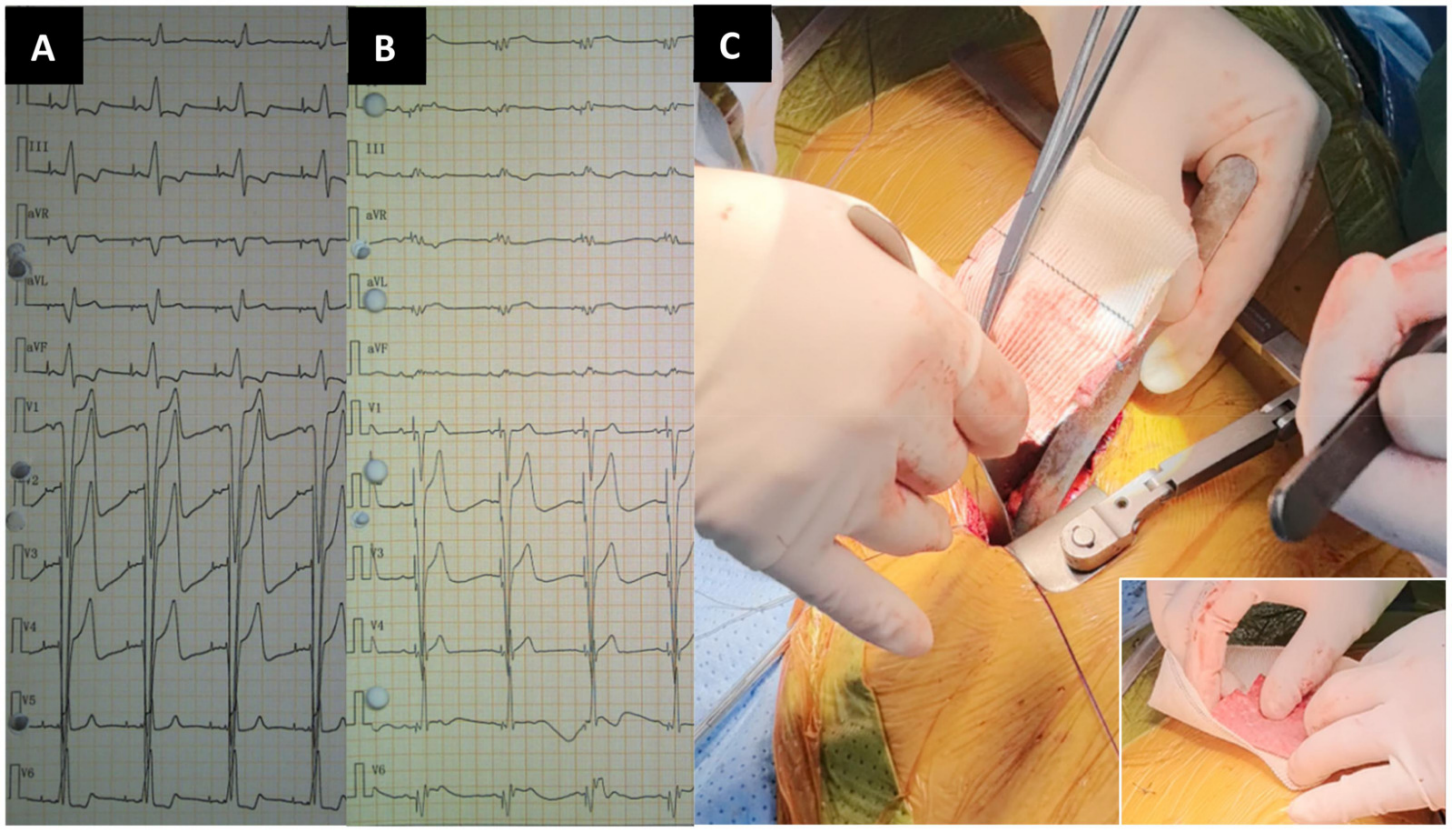
**(A)** ECG before CRT upgrade. **(B)** ECG after CRT upgrade. **(C)** The large picture shows artificial blood vessels wrapped around folded polyester patches and sewn together as one. The small picture at the lower right corner: The upper red material was a folded polyester patch with blood stains, and the lower white material was an incised artificial blood vessel.

A 68-year-old male patient was admitted to the outpatient clinic due to recurrent dyspnea after CRT implantation. The patient underwent dual-chamber pacemaker implantation for bradycardia. Battery depletion and heart failure (HF) occurred 8 years after operation, and CRT was upgraded. After CRT upgrade, the QRS duration was shortened from 200 ms to 168 ms (Figures 1A, B). PNS occurred 2 weeks after CRT. PNS could not be eliminated by increasing pulse width or reducing pacing output. Eventually, CRT was turned off due to intolerance to PNS.

**Figure 2:**
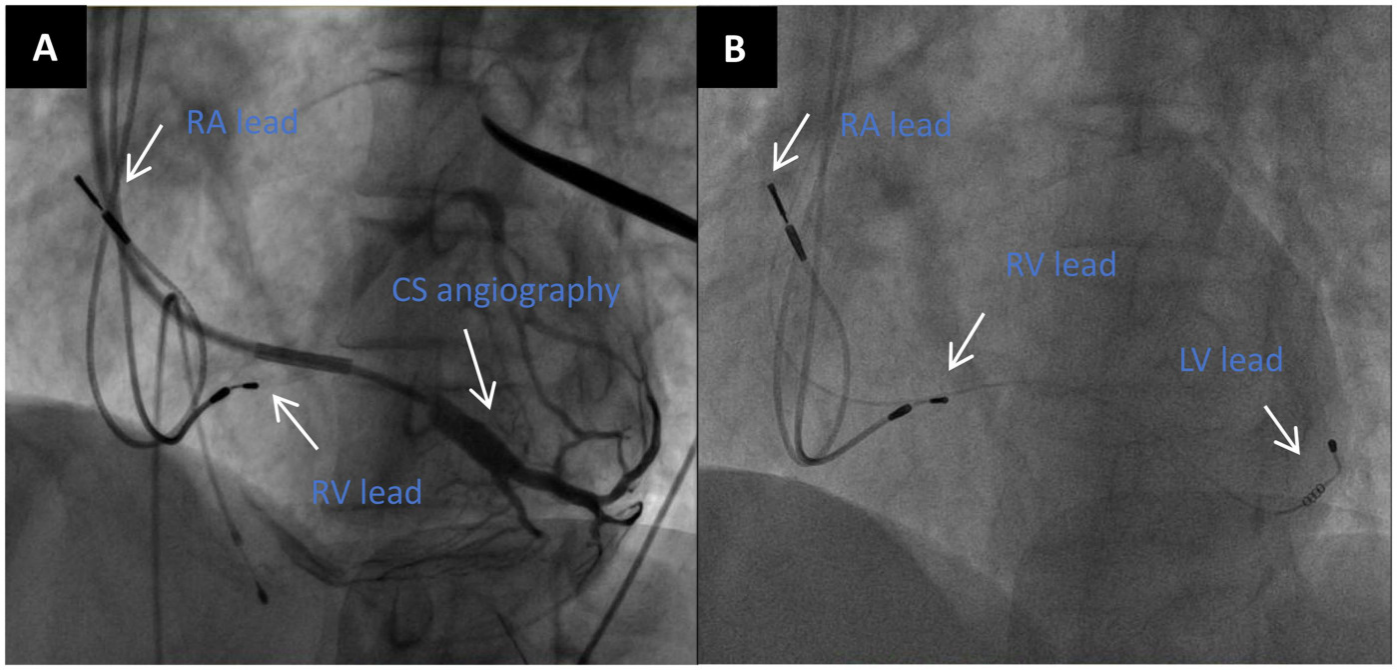
**(A)** LAO 45° CS angiography. **(B)** LAO 45° view showed the positions of the right atrial(RA) lead,right ventricular(RV) lead and LV lead.

Therefore, reactivating the CRT system or eliminating the PNS becomes particularly crucial. The medical team initially considered electrode resetting, but reviewed the patient's CRT surgical images, and there was no better CS target vessel to replace (Figure 2). When the patient underwent CRT upgrade, severe stenosis had already occurred in the left subclavian vein. Based on the above reasons, it was very difficult to reset the electrode for this patient. If the electrode was reset, the patient's pacemaker pocket would be opened for the third time, and the risk of pocket infection was high.

**Figure 3:**
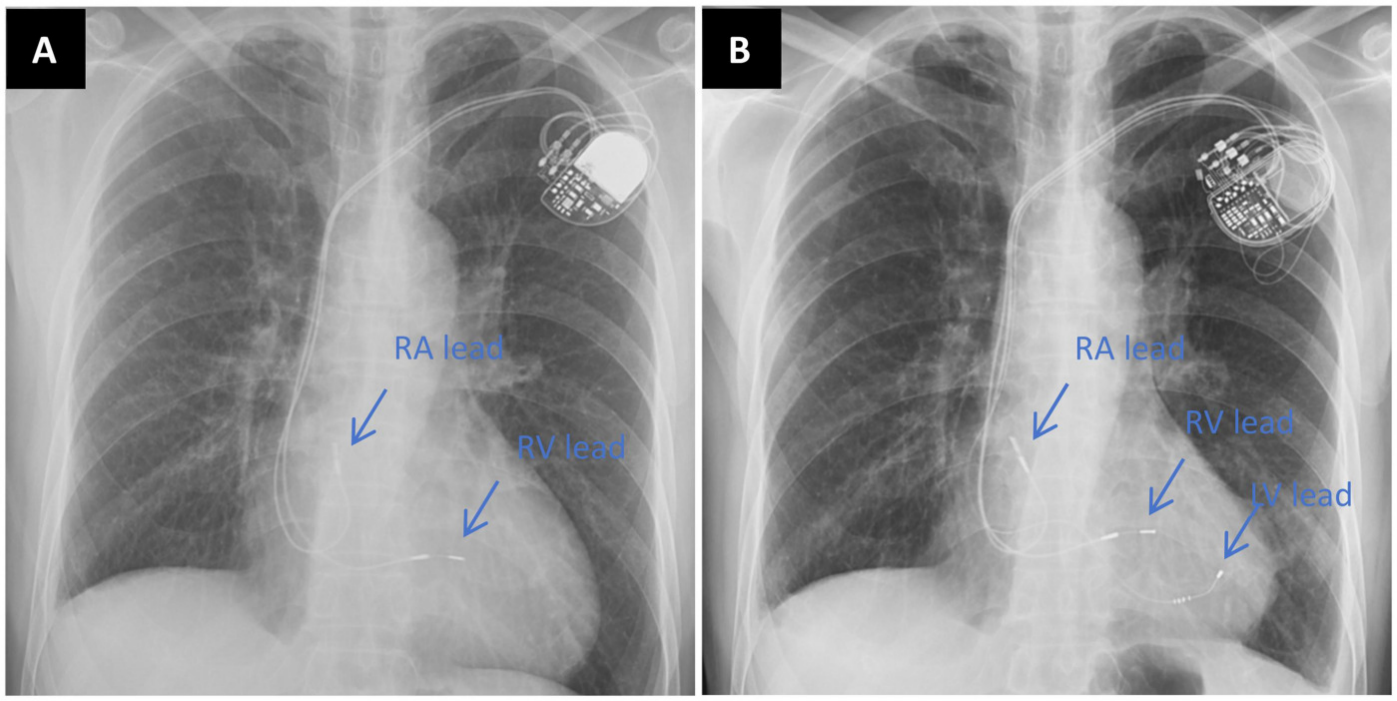
**(A)** chest radiograph before CRT upgrade. **(B)** Chest radiograph 1 year after CRT upgrade.

Based on the above situation, our cardiac team performed surgical PN isolation for this patient without affecting the effect of CRT super response. Under general anesthesia with tracheal intubation and off-pump, a small incision (about 4-5 cm) was made through the left 4/5 intercostal space to enter the left thoracic cavity, and the pericardium was cut above the left PN. The Hemashield platinum woven double velour vascular graft (M00202175428P0, Intervascular SAS, France) wrapped with polyester patch (DFS-P-1010 felt-type, Chester, Shanghai) (Figure 1C) was sutured and fixed with the parietal pericardium, and the LV electrode and the left PN were isolated. The PNS threshold was significantly increased during the operation. The preoperative PNS thresholds of the patient was 1.0 V/1.0 ms (myocardial threshold 1.5 V/1.0 ms) and the postoperative improvement was 6.0 V/1.0ms. The patient recovered well and discharged from hospital after operation. One year later, chest radiograph demonstrated reduction in cardiac size (Figure 3). Up to the present follow-up, the patient has not experienced recurrent PNS.

## Discussion

Among patients with reduced ejection fraction, widened QRS complex, and New York Heart Association (NYHA) class II to IV heart failure, CRT reduces the rate of HF rehospitalization and all-cause mortality (1, 2). Nevertheless, more than 30% of patients remain non-responsive to CRT. Conduction system pacing (CSP) has advanced rapidly in recent years. It may be considered as an alternative in patients with CS anatomical anomalies, intolerance to PNS, lead dislodgement, CRT non-response, or high pacing thresholds (3). In selected patients, CSP may yield superior efficacy compared with CRT. Conventional CRT still holds advantages in patients in whom CSP fails for various reasons. If all transvenous implantation attempts are unsuccessful, left ventricular epicardial pacing represents a viable alternative option.

PNS is a frequent complication following CRT. Usually, we can try to eliminate PNS by changing the pacing threshold, increasing the pulse width, and changing the pacing polarity. The LV electrodes implanted in the patient were the earlier Medtronic 4195 electrode, which was a unipolar electrode. The probability of PNS occurrence was significantly higher than that of bipolar and quadripolar left ventricular lead. Once PNS occurs, it is difficult to eliminate it by external programming (4). In this patient, PNS emerged shortly after CRT implantation. Lead displacement was ruled out by chest radiography. PNS was related to postural variation: absent in the supine position but evident in the sitting position. Intraoperative PNS testing has limitations and may not accurately reflect real-world daily activity.

If PNS can not be eliminated by external programmed control, we should consider resetting electrodes, such as using quadripolar LV lead, CSP, epicardial electrode and so on. The quadripolar LV lead can eliminate most of the occurrence of PNS by postoperative programming and avoid electrode reset, but there are exceptions (5, 6). Unfortunately, the patient developed PNS in 2016, and quadripolar LV leads were not available in China at that time, nor was left bundle branch pacing(LBBP). In addition, there were venous access and target vessel problems. Although right-sided His bundle pacing(HBP) could completely avoid PNS, it was technically difficult and was also associated with an increased threshold in the later stage. Epicardial pacing could not guarantee no recurrence of PNS in the later stage. If the pacemaker sac of the patient was opened for the third time, the risk of sac infection also needs to be considered. Once it occurs, the consequences would be severe. For the above reasons, the patient did not undergo electrode reset.

After discussion by the cardiac team and informed consent of the patient, we performed surgical PN isolation using a combination of artificial blood vessel and polyester patch. It is worth noting that at the beginning, we simply used the polyester patch to fold and isolate PN, and PNS disappeared immediately, but PNS appeared again after more than ten minutes. It might be due to the conductivity of the polyester patch after being wetted by blood. The polyester patch was wrapped with artificial blood vessels and sutured into one piece, then fixed to the pericardial parietal layer. The effect was good, and no PNS was observed after 40 to 50 min. The operation was successfully completed. Jin-Long Huang et al. (7) achieved similar outcomes in an intact swine heart model. PNS was avoided by placing a PTFE patch over the LV lead or a graft around the PN. However, only intraoperative results were available and follow-up was not possible. Due to successful elimination of PNS, the CRT function was restored, At 2 years after operation, LVEDD was reduced from 7.0 cm before CRT upgrade to 4.5 cm, and LVEF was increased from 30% to 50%. The patient was followed up for 10 years, with no recurrence of PNS.

This paper discusses the treatment of PNS after CRT, and we propose a new method to eliminate PNS. Although this method is invasive, it has the advantages of simple material and definite curative effect. For refractory PNS after CRT, this method can be considered if you cannot reset the electrode for various reasons.

It should be clarified that the patient described in this manuscript is a completely independent and distinct patient, with no overlapping data or duplicated content with the case reported in our related manuscript published in Frontiers (ID: 1762109).

## Data Availability

The original contributions presented in the study are included in the article/Supplementary Material, further inquiries can be directed to the corresponding author.
